# Thank You to Our Reviewers

**DOI:** 10.1097/MD.0000000000005741

**Published:** 2016-12-30

Thank You to Our Reviewers

The *Medicine*^*®*^ Editorial Office would like to thank all of our expert volunteers for their continued devotion and generosity to the journal. Peer review is an essential part of publishing high quality content and their contributions and feedback are fundamental to the process.

As a sign of our appreciation we want to recognize all of the reviewers who contributed to the journal in 2016. To all of our reviewers, we are grateful for your contributions and look forward to working with all of you again in 2017!

Best Regards,

Tom Pacific

Publisher

This is an open access article distributed under the Creative Commons Attribution License 4.0 (CCBY), which permits unrestricted use, distribution, and reproduction in any medium, provided the original work is properly cited.

Alvaro Abarzua

Edson Abdala

Mohamed Abdalaal

Ahmed Abdel-Razik

Babak Abdinia

El Banna Abduh

Sheik Abdul Khadir

Koichiro Abe

Ludovico Abenavoli

Abhishek Abhishek

Vered Abitbol

Khaled Abu Ali

Ethem Acar

Jeanne Ackman

Hisashi Adachi

Charlotte Adamczick

Mariusz Adamek

D.Cory Adamson

Jeremy Adler

Sanjit Agarwal

Eva Ageberg

Shagun Aggarwal

Mukta Agrawal

Archi Agrawal

Ritesh Agrawal

Sarah Ahmadi-Erber

Armin Ahmed

Seong Joon Ahn

Hiroyuki Aihara

Akash Ajmera

Sami Akbulut

Tomi Akinyemiju

Shintaro Akiyama

Faruk Aksoy

Tolga Aksu

Recep Aktimur

Graciela Alarcón

Nadia Alatrakchi

Dara Albert

Neville Alberto

Mohamed Alboraie

Ragheed Al-Dulaimi

Marianna Alesi

Lauren Alexander

Ayman Alghoraieb

Walid Al-Ghoul

Wael Alkhiary

Krumholz Allan

Pierre Allemann

Benito Almirante

Erkan Alpsoy

Kawther Alquadan

Terje Alraek

Khalid Al-Rubeaan

Fatima Alshbool

Sattar Alshryda

Firas Al-Taweel

Servet Altay

Bülent Altunoluk

Alessia Alunno

Julio Alvarez-Pitti

Ivan Alvarez-Twose

Venancio Alves

Giordano Alves

Marco Alves

Silvana Alves Pereira

Amjad Alwaal

Saeed Alzghari

Oluwatoyin Ameh

Mohamadreza Amiri

Michele Ammendola

Somchai Amornyotin

Encarnita Ampil

Anne Amstrup

Mingrui An

Jian An

Seong Soo An

Young-Sil An

Nikolaos Anagnostou

Lakesh Anand

Ljubica Andersen

Ethan Anderson

Giuseppe Andò

Antonio Andreacchio

Fodor Andrei

Kristoffer Andresen

Sweta Andrews

Ioannis I Androulakis

Wes Angel

Fabio Angeli

Andrea Angelini

Gougelet Angelique

Thomas Angelovich

François Angoulvant

Sabiha Anis

Ravindran Ankathil

Giuseppina Annicchiarico

Jonathan Anolik

Nagaraju Anreddy

Wahid Ansari

Alessandro Antonelli

Anastasia Antoniadou

Graziano Antonino

Zheng Ao

Mahmut Arabul

Hiromasa Arai

Edith Arany

Paolo Arcidiacono

Rosaria Arcone

Sara Arévalo

Takaaki Arigami

Ingrid Arijs

Kazuhiko Arima

Kimiyoshi Arimura

Reiko Arita

Dr Pedro Armario

Joel Aronowitz

Javier Arpa

Mohamed Arruj

Ismail Arslan

Mehmet Artaç

Nibal Arzouni

Enrique Asensio Lafuente

Muhammad Asif

Jaya Asirvatham

Renata Assis

Lambert Assoumou

Nelson Astur

Ángel Asúnsolo

Funda Atamaz

Esra Erkoç Ataoğlu

Panagiotis Athanasopoulos

Greg Atkinson

Dalia Attallah

L Atzori

Laurent Auboire

D Aune

Riccardo Autorino

Christopher Auvenshine

Alberto Avolio

Alfonso W Avolio

Sara Awad

Reda Awali

Constantine Axiotis

Yusuf Aydemir

Mesut Aydin

Mesut Ayer

Olugbenga Ayodele

Walid Ayoub

Seham Azab

Shafquat Azim

Kosuke Azuma

Hanan Azzam

Zarko Babic

Veronika Bachanova

Hagen Bachmann

Konstantinos Bachoumas

Parisa Badiee

Raffaele Badolato

Younghyeon Bae

Alan Baer

Sabrina Bagaglio

Shashwatee Bagchi

Puneet Bagga

Mathias Bähr

Kunhao Bai

Ya Na Bai

Robert Bailey

Anamika Bajpai

Sule Mine Bakanay

Ioannis Bakolis

Kishan Bakrania

Mohamed Bakri

Mehmet Akif Baktir

Chandrashekhar Bal

Daniel Vasile Balaban

Uthra Balaji

Chiara Baldini

Mehmet Balioglu

Rajesh Balkrishnan

Javier Ballesteros

Stefano Ballestri

Gianpaolo Balzano

Christina Bamia

Maciej Banach

Eugeniu Banu

Renyue Bao

Xuhui Bao

Katherine Baquerizo Nole

Michal Barak

Gocha Barbakadze

Giovanni Barbanti Brodano

Mustafa Barbhuiya

Franca Barbic

Fabio Barbieri

Robert Barkin

Satish Prasad Barnawal

Bruno Baroni

Elsie Baronia-Locson

Ellis Bartlett

Francesco Bartoli

Elena Bartoloni Bocci

Sibel Basaran

Debasish Basu

Sebahat Başyiğit

Ibrahim Batal

Kiran Batra

Maurizio Battaglia Parodi

Maxime Battistella

Hale Batur Çağlayan

Ralf Bauer

Hélène Bauer

Mathias Baumert

Jagadeesh Bayry

Shabnam Bazmi

David Bearden

Elisardo Becoña

A Bektas

Derek Bell

Teresa Bell

Silvia Bellando Randone

Fernanda Bellodi Schmidt

Abdelouahab Bellou

Telmo Augusto Belsuzarri

Robert Belvis

Raoul Belzeaux

Touria Benamar

N. Benito

Julián Benito-León

Roelof Bennink

Vasili Berdoukas

Janneke Berecki-Gisolf

Nancy Berliner

Jose Luis Bermejo

M Rosa Bernal-Lopez

Nicole Bernard

Boris C. Bernhardt

Da Berntsen

Massimiliano Berretta

Carolyn Berryman

Ines Bersch

Gaetano Bertino

Federico Bertuzzi

Jennifer Berumen

Ahmet Besir

Huseyin Besiroglu

Feyzullah Besli

R Beurskens

Jinendra Bhagat

Kalyan Ram Bhamidimarri

Tattwamasi Bharadwaj

Girish Bhatt

Sanjeev Bhoi

Lokesh Bhushan

Xiaofang Bian

Chunjing Bian

Bianba Bianba

Bianca Bianco

Antonino Bianco

Zafer Bicakci

Thomas Bice

Thomas Biedermann

Jean Joel Bigna

Babitha Bijin

Jawad Bilal

Shymaa Bilasy

Ugur Bilge

Orhan Binici

Giuseppe Biondi-Zoccai

Stephen Birch

Mark Birkenbach

Massimiliano Bissolati

Nidhan K. Biswas

Petter Bjornstad

Jan Blaha

Julià Blanco

Mona Boaz

C-Thomas Bock

Erika Boesen

U Boggi

Zdzislaw Bogucki

Krzysztof Bojakowski

Doron Boltin

Laura Bonanni

A Bonifaz

Stefanos Bonovas

Laura Boomer

Peter Boor

Diana Boraschi

Gerardo Bosco

Jean-David Bouaziz

Brian Boulay

Alain Boussuges

Anders Boyd

Laurent Boyer

L. Boyer

Mark Bradov

Virginia Brady

Parag Brahmbhatt

Paulo Henrique Braz-Silva

Irena Brčić Karačonji

Lars Breimer

Morten Breindahl

Gary Brennan

Charles Brennan

Lm Brewster

Matthew Brothers

Matthijs Brouwer

Jeremy Brownstein

Norbert Brueggemann

Joseph Brugada

Paolo Brusini

Xiangning Bu

Catharine Buchan

Matthias Büchter

Ulrike Buchwald

Aaron Buckland

Benjamin Buecking

Robin Buerki

Marco Bueter

Jack Buhrow

Marwan Bukhari

Daniel Bunout

Carlo Buonerba

David Burger

Ruben Burgos-Vargas

Harry Burke

Raquel Burrows

Huseyin Buyukgol

Fernanda Cabral

Orazio Caffo

Zhen Cai

Anping Cai

Zhiyou Cai

Dachuan Cai

Weijia Cai

Zhi-Gang Cai

Guangyan Cai

Sanjun Cai

Yong Cai

Yihua Cai

Gianluca Caiazzo

Juan Caicedo

Saverio Caini

Murat Cakir

Anna Rita Calavalle

Andrea Calcagno

Mukadder Calikoglu

Priscilla Callahan-Lyon

Yvon Calmus

Cristina Calvo

Juan Camacho

Miguel Camafort-Babkowski

Luciana Aparecida Campos

Ozgur Can

Ali Canbay

Rodrigo Canellas

Pietro Canetta

Anna Cantarutti

Xu Cao

Kaixiang Cao

Lu Cao

Muqing Cao

Huojun Cao

Jia Cao

Huijuan Cao

Zhijuan Cao

Ping Cao

Margherita Capasso

Ezio Cappello

Franco Capsoni

Kenneth Cardona

Claudia Cardoso

Filipe Cardoso

Teresa Cardoso

William Carey

Marco Carotenuto

Patricia Carreira

Pricivel Carrera

Luis Carvajal Carmona

Elaine Carvalho

Ana Carvalhosa

Jose Casado

Marco Cascella

Antonella Castagna

Rogerio Castilho

Luis Manuel Castillo

Spero Cataland

Meghan Caulfield

Giulia Martina Cavestro

Alessandro Cavicchioli

Mario Cazzola

Osman Cekic

Onur Celik

Jelena Celutkiene

Candemir Ceran

Giuseppe Ceraudo

Selma Cernea

Furkan Certel

Ricard Cervera

Carlos Cervera

Ali Cetinkaya

Alejandro Chade

Weiran Chai

Yoram Chaiter

John Chambers

Lung Chan

Kun-Ming Chan

Kwan-Leung Chan

C.-C. Chan

Derek Chan

Tommy Chan

Samudi Chandramathi

Chia-Chu Chang

Yi-Cheng Chang

Hong Chang

Shih-Lun Chang

Ke-Vin Chang

Kai-Po Chang

Won Seok Chang

Hsueh-Wei Chang

Shih-Liang Chang

Jeffrey Chang

Yu-Ting Chang

Deng Changwen

Chien-Ming Chao

Chia-Ter Chao

Tsu-Yi Chao

Shu-Ping Chao

Ana Charrua

Indranil Chattopadhyay

Nikolaos Chatzizacharias

Rashmi Chaudhary

Rahul K Chaudhary

Akhilanand Chaurasia

Ken Chavin

Suhash Chavva

Sanjeev Chawla

Saurabh Chawla

Yen Chee

Robert Chen

Weina Chen

Demeng Chen

Xiang Chen

Xiaoping Chen

Haoyu Chen

Guangming Chen

Chung-Yu Chen

Xin Chen

Hairong Chen

Lin Chen

Xiukai Chen

Chengpeng Chen

Kuo-Hu Chen

Hao Chen

Lili Chen

Zhonglei Chen

Yalei Chen

Hua Chen

Chyi-Liang Chen

Huey-Yi Chen

Min Chen

Guo Chen

Yu-Wei Chen

Xiao-Yun Chen

Qing Chen

Tenghui Chen

Xiabin Chen

Zirong Chen

Dongshi Chen

Hsing-Yu Chen

Chien-Hung Chen

Jiamin Chen

Jun Chen

Ji-Yih Chen

Jiajia Chen

Limin Chen

Mengshi Chen

Zijiang Chen

Xin-Zu Chen

Youjun Chen

Wei Chen

Shan Chen

Yen-Shan Chen

Chen Chen

Zhenguang Chen

Dong-Yi Chen

Fei Chen

Chong Chen

Lucy Chen

Margaret Chen

Jiajian Chen

Shih-Ann Chen

Carl Chen

Chih-Chung Chen

Huafu Chen

Li-Da Chen

Su-Fang Chen

Gang Chen

Chien-Ho Chen

Yung-Tai Chen

Yihe Chen

Pei-Lung Chen

Sheng Chen

Jianding Cheng

Binbin Cheng

Po-Ching Cheng

FJ Cheng

Lijun Cheng

Pin-Nan Cheng

Tain-Junn Cheng

Bi-Hua Cheng

Han-Yi Cheng

Mu-Hua Cheng

Kam Chau Cheng

Ching-Feng Cheng

Hua Cheng

Chen-Hwan Cherng

Pavel Chernyavskiy

Yun-Chung Cheung

C.W. Cheung

Pui Yee Cheung

Sumedha Chhatre

Chih-Yu Chi

Chi-Ling Chiang

Jiun-Yang Chiang

Priyadarshini Chidambaram

Chih-Yen Chien

Francesca Chiodi

Hung-Yi Chiou

King-Wah Chiu

Calvin Chiu

Lechoslaw Chmielik

Steven Chmura

Hee Young Cho

Joo Young Cho

Na-Eun Cho

In Rae Cho

Young-Keol Cho

Arun Chogle

Hoon Young Choi

Jun Yong Choi

Jae-Mun Choi

Joon Young Choi

Jong Won Choi

Yoonyoung Choi

Suk Chon

Young Eun Chon

Ching Ning Chong

Kun-Ta Chou

Yiing-Jenq Chou

Shreyasi Choudhury

George Chouliaras

Pratim Chowdhury

Xiaogang Chu

Chia-Yu Chu

Lee-Ming Chuang

Chih-Hsien Chuang

Ting-Wu Chuang

Shao-Yuan Chuang

Yu-Chung Chuang

Fu-Tsai Chung

Chia-Min Chung

Ej Chung

Hyun Chul Chung

Hyun Woo Chung

Mei Chung

Chen-Chih Chung

Vincent Ch Chung

Quirino Ciampi

Michele Ciccarelli

Arrigo Cicero

Yasin Cinar

Abdullah Cingü

Maria Leticia Cintra

William Clafshenkel

Isabelle Cleynen

Alessandra Codegone

Eduardo Coelho

Bernard Cohen

Ohad Cohen

Jan Willem Cohen Tervaert

Matthew Cohn

Amparo Coira

Julio Collazos

John Collins

Didem Colpan Oksuz

Giacomo Colussi

Lucieni Conterno

Aashish Contractor

Alan Cook

Mark Cookson

Michele Coppola

Gino Roberto Corazza

Graziamaria Corbi

Christian Cordano

Divi Cornec

Catherine Cornu

Salih Coskun

Gabriela Costa

Cecilia Costiniuk

Mª- Luz Couce

Marion Couch

Ilker Coven

Megan Crane

Paulo Criado

Stefano Crippa

Michael Cripps

Bruce Crookes

Cynthia Crowson

Ivana Cruz

Gokhan Cuce

Ferhat Cüce

Yi Cui

Liyang Cui

Baojuan Cui

Li-Gang Cui

Gabriel Culbert

Daniel Culver

Valdo José Dias Da Silva

Marta Dąbrowska

M Dąbrowski

Konstantinos Dafopoulos

Wendy Dahl

Menghua Dai

Zhi-Jun Dai

Chia Yen Dai

Xiaohua Dai

Hui Dai

R Dajani

Fabrizio Dal Farra

Peter Daley

Cher Dallal

Josep Dalmau

Felipe Daltoé

Andrew Daly

Archana Damle

Giancarlo D’Andrea

Cem Dane

Yonghui Dang

Yalong Dang

Yifang Dang

N Danışman

Ann Danoff

Nihaya Daoud

Sayed Daoud

Alexandros Daponte

Giovanni Dapri

Undurti Das

Deepika Sharma Das

Greta Luana D’Ascoli

Soumit Dasgupta

J Dasta

Ilker Dastan

Fereydoun Davatchi

Weaver David D.

Sonal Dayama

Martin De Borst

Marco Antonio De Carvalho-Filho

Benito De Celis

Stefano De Cillà

Steven De Decker

Luc De Haro

Javier De La Fuente Aguado

Raffaele De Palma

Valli De Re

Francesco De Rosa

Salvatore De Rosa

Roberta De Rosa

Luca De Santo

Alfonso De Stefano

Carlos De Vasconcelos

Rebecca Deal

Ralph Debiasi

Angelica Debs

Anastasia Dede

Takashi Dejima

Victoria Delgado

Luciano Delgado-Plasencia

Mario D’Elios

Dionysios Dellaportas

Marika Dello Strologo

Atakan Demir

Lütfi Demir

E Demirkaya

Suleyman Demiryas

Xuefeng Deng

Kai Deng

Hao Deng

Ruishu Deng

Zhengrong Deng

Guohong Deng

Jiong Deng

Pieter Depuydt

Mohammad Derakhshan

Fatma Derbali

Benoit Dervaux

Tanvi Desai

Ediriweera Desapriya

Raziye Desdicioglu

Samuel Deshayes

Sachin Kumar Deshmukh

Anindya Dey

Mahua Dey

Fu Deyuan

Sofie Dhaese

Sandeep Dhall

Neeraj Dhaun

Jin Di

Paola Di Carlo

Giuseppe Di Costanzo

Roberto Di Donato

Angelo Di Iorio

Vito Di Lernia

Giuseppe Di Lorenzo

Michele Di Martino

Rocco Di Mascio

Luigi Di Serafino

Rossella Di Trolio

Giovanni Di Zenzo

Smaranda Diaconescu

Xingxing Diao

Emilia Diego

Francesco Dieli

Markus Diener

Max Dieterich

Konstantinos Dimas

George Dimitriadis

Johannes C. Athanasios Dimopoulos

Qiliang Ding

Kefeng Ding

Wenyuan Ding

Liya Ding

Dale Ding

Juan Ding

Xiaoyan Ding

Daniel Dirkmann

Susanthy Djajalaksana

Jun Young Do

Richard Do

Ekaterina Dobryakova

Carine Domenech

Philippe Domenech

Ana-Marija Domijan

Lars Donath

Zhao Dong

Jia-Hong Dong

Hongmei Dong

Lei Dong

Junchao Dong

Maria Pina Dore

Deepak Doshi

Richard Doty

Yuetan Dou

Marcelle Dougan

Evangelia Dounousi

Paulo Dourado

Periklis Dousdampanis

Esther Dr. Saguil

Luciano Drager

Michel Drancourt

Johann Dréanic

Yang Du

Peng Du

Li Du

Jun Du

Xiaoqiong Duan

Lihua Duan

Sergio Duarte

Catherine Dube

Jennifer Duchon

Anne-Sophie Ducloy-Bouthors

Ender Dulundu

Donald Dumford

Mehmet Akif Dundar

Madeleine Durand

Emanuele Durante-Mangoni

Jacob Ecanow

Lydia Eccersley

Sarah-Jayne Edmondson

Fahad Edrees

Rachael Edwards

Emmanuel Effa

Jimmy Efird

Tasnim Eghbal Eftekhaari

Brian Egleston

Edvard Ehler

Elana Ehrlich

Thomas Eigentler

Rasha El Etreby

Mohamed El Kassas

Mohamed El Sorogy

Reza Elahimehr

Mohssen Elalfy

Mohja El-Badawy

Nikolas Eleftheriadis

Samar El-Ganainy

Deilson Elgui De Oliveira

Yeung Ella

Adrian Elliott

Mohamed El-Nady

Ahmed Elshamy

Mohammed Elsheemy

Abdelhalim Elsherif

Hussien Elsiesy

Theophilus Emeto

Dario Englot

Jae Seon Eo

Emily Erbelding

Barbaros Erdal

Mehmet Erdoäÿan

Serpil Erdogan

Akca Toprak Ergonen

Maury Eric

Ahmet Eroglu

Cem Ertan

Shankar Esaki Muthu

Güneş Esendağlı

Ahmet Emre Eskazan

Isaac Estevan

José Carlos Esteves Veiga

Joy Evangelista

Carlton Evans

Osayande Evbuomwan

Charles Everett

Mohamed Fadl Elmula

Riccardo Maria Fagugli

Benjamin Fairfax

Vicenç Falcó

Marco Falcone

Mercé Falip

Joe Falkinham

Mahdi Fallah

Mohammad Kazem Fallahzadeh

Luca Faloppi

Yu-Chen Fan

Shengjun Fan

Zhenqian Fan

Longchang Fan

Chao Fan

Yang Fan

Run Fan

Lin Fan

Jia Fan

Dongwei Fan

Francesco Fanfulla

Bin Fang

Aiqin Fang

Chao Fang

Gang Fang

Meiyu Fang

Alberto Farias

Daniel T Farkas

Sanja Farkas

Gyula Farkas Jr.

Timothy Farley

Fabrizio Favales

Raphael Favory

Iman Fawzy

Marchlinski Fe

Eniko Feher

Eva Feigerlova

Yuri Feito

Zhanhui Feng

Ye Feng

Chenchen Feng

Yi Feng

Wei Feng

Shi-Qing Feng

Jean-Paul Fermand

Andreu Fernandez-Codina

César Fernández-De-Las-Peñas

Nuria Fernández-Hidalgo

Alberto Fernandez-Medarde

Cristina Fernández-Pérez

Joana Ferreira

Pablo Ferrer

Clodoveo Ferri

Matteo Ferro

Ondrej Fiala

Marco Fichera

Thalia Field

Michele Figus

Pierre Filhine-Tresarrieu

Geraldo Lorenzi Filho

Vassilis Filiopoulos

Jennifer Fillaus

Sıddıka Fındık

Antoine Finianos

Jodok Fink

Taylor Finseth

Mathieu Fiore

Paolo Fiorina

Giulia Fiorini

Bonnie Firestein

Bani-Sadr Firouzé

Marc-Olivier Fischer

Marc Fischler

Lauren Fishbein

Timothy Fitzgibbons

J M Flack

Martin Floer

Shannon K. Flood-Nichols

Efren Flores

Paolo Fogagnolo

Guillaume Fond

Shirley S.M. Fong

Ricardo Fontes-Carvalho

Jia Nee Foo

Chee Yoong Foo

M. Foppa

Ricardo Raul Forastiero

Heide Ford

Dr. Steven Forland

Alan Fotoohi

Daniel Fouladi

Daryl Fourney

Pierre Fournier

Irene Frachon

Rodrigo Fraga-Silva

Camille Frances

Raffaella Franciotti

Davide Francomano

Justin Fraser

Michael Frass

Thomas R. Frieden

Ana Belén Friginal-Ruiz

Stephanie Fritz

Luca Frulloni

Cheryl Frydman

Yonglun Fu

Yue Fu

Bishi Fu

Ting Fu

Tomoko Fujii

Yutaka Fujiwara

Shoichi Fukui

Michiaki Fukui

Geoffrey Funk

Massimo Fusconi

Yasutaka Fushimi

Daniel Fuster

Edward Fysh

Patrik Gabikian

Manila Gaddh

Yunchao Gai

Bhavin Gajera

Georgios Gakis

Olimpi Galasso

Norman Galbreith

Britta Galling

Camila Gallo

Juan Galvez

Revital Gandelman-Martin

Ashish Gandhe

Sara Gandini

Shobana Ganesan

Zhongli Gao

Linbo Gao

Chun Gao

Chunxu Gao

Xin Gao

Tian Gao

Chenxi Gao

Dengfeng Gao

Lan Gao

Jian Dong Gao

Xiang Gao

David Garcia

Christian Garcia

Iñaki Garcia De Gurtubay

Xavier García-Albéniz

David García-Azorín

Santiago Garcia-Lopez

Xavier García-Massó

Sara Garcia-Ptacek

Laurent Garderet

Chris Gardiner

Clarice Gareri

Giuseppe Gargiulo

Venkata Garikipati

Marcio Garrison Dytz

Thomas Gary

Wambui G. Gathirua-Mwangi

Stéphane Gaudry

Umair Gauhar

Maria Gavriatopoulou

Ryszard Gawda

Maria Gazouli

Diego Gazzolo

Ning Ge

Habip Gedik

Michael Gelfand

Jiafeng Geng

Simonetta Genovesi

Sophie Georgin-Lavialle

Ekavi Georgousopoulou

Anjali Gera

Roberto Gerli

Hayley Gershengorn

Sumantra Ghosh

Roberto Giacomelli

Vania Giacomet

Riccardo Giampieri

Kleanthis Giannoulis

Vasileios Giapros

Brent Gibbons

Olivier Gié

P Gifford

M. Esther Gil-Alegre

Nicolas Girerd

Giampiero Girolomoni

Paolo Gisondi

Mario Giuffrè

Antonio Giuliani

Ioannis Gkegkes

Evan Glazer

Michael Glocker

Michael Gluth

Se-Il Go

Amede Gogovor

N Simay Gökbayrak

Stanley Goldfarb

Daniel Goldsmith

Delia Goletti

Carlos Gómez-Escalonilla

Isabel Gomez-Hurtado

Jucier Gonçalves Júnior, Jr

Pedro Augusto Gondim Teixeira

Qi Gong

Yan Gong

Wei Gong

Chang Gong

Michel Gonzalez

Luis Gonzalez

Humberto Gonzalez

Sergio Gonzalez Bombardiere

Luis Alonso González Naranjo

Miguel Gonzalez-Gay

Nicolas Goossens

Quinton Gopen

Vijayaprasad Gopichandran

Bicanic Goran

Miguel Gorgolas

Tahsin Görgülü

Nandu Goswami

Lucy Goulding

Nelia Gouveia

Usha Govindarajulu

Urs Granacher

Michael Grant

Elena Grebenciucova

Michael Green

David Greenblatt

Sr Greisen Greisen

Andrew Griffith

Andrea Grillo

Konstadina Griva

Lukasz Grochola

Nynke Groenewold

Dustin Grooms

Ehud Grossman

Sandeep Grover

Anete Grumach

Stephen Grupke

Xiang Gu

Zhimin Gu

Wan-Jie Gu

Haiyong Gu

Ying Gu

Xuehai Guan

Hanfeng Guan

Giovanni Guaraldi

Angel L. Guerrero

Luiza Guilherme

Carlos Guillen Astete

Hélio Penna Guimarães Guimarães,

Pierre Guinot

Ilker Gul

Ibrahim Guler

Simone Gulletta

Esra Gunduz

Metin Gündüz

Marianne Gunell

Alime Gunes

Sezgin Güneş

Shuang Guo

Xunyang Guo

Hao Guo

Shicheng Guo

Zhen-Ni Guo

Hongyan Guo

Hui Guo

Wei Guo

Wenbin Guo

Xiuhua Guo

Xiaomao Guo

Shi-Bin Guo

Qi Guo

Rajesh Gupta

Seema Gupta

Sachin Gupta

Bal Kishan Gupta

Raavi Gupta

Anil Kumar Gupta

Nitin Gupta

Rishein Gupta

Sk Gupta

Samir Gupta Md

Mustafa Gursoy

Simona Gurzu

Ana Gutiérrez

Tae-Geun Gweon

Georg Györi

Felix Hüttner

Mark Haas

Peiman Haddad

Dieter Haemmerich

Batoul Haerian

Yoshio Haga

Katrina Hagberg

Mahdi Haghighatafshar

Ajay Hailder

Bereketeab Haileselassie

K Haldorsen

Braden Hale

Heidemarie Haller

David Hallman

Rola N. Hamam

Hidetaka Hamasaki

Mette Hambraeus

Amir Ali Hamidieh

Mohsen Hamidpour

John Hammond

Zhongji Han

Xiaomei Han

Seung Hyeok Han

Bing Han

Saskia Handari

Hu Han-Hwa

Guang Hao

Jianyo Hao

Weimin Hao

Masaki Hara

Yusra Harahsheh

Murat Harputluoglu

Peter Harri

Elizabeth Harris

David Hart

Marek Hartleb

Hajime Hasegawa

Kosuke Hashimoto

Monika Hasnat

Jaythoon Hassan

Behrooz Hassani-Mahmooei

Lewis Hassell

Ibrahim Hatemi

Emmanouil Hatzipantelis

Raphael Hau

Peter Havens

Shannon M. Hawkins

Yi He

Zhiheng He

Jing He

Fanggang He

Hua He

Yang He

Jin He

Yu He

Xiang He

Jian He

D He

Qing He

Lee Hebert

Refaat Hegazi

Hauke Heinzow

Ryan Helmick

Andrés Henao-Martínez

Randolph Hennigar

Ji Haeng Heo

Frank Herbstreit

Theresa Herdeiro

Yair Herishanu

Leal Herlitz

Julio Hernandez

Guillermo Herrera

Kaisa Hervonen

Ingrid Heuch

Mate Hidvegi

Saima Hilal

Corrilynn O. Hileman

Tom Hill

Takahisa Hiramitsu

Tzong-Shiann Ho

Chung-Han Ho

Robin Ht Ho

Gerhard Hobusch

Thomas Hocker

Christopher Hocking

Stefan Hohaus

Samuel Hohmann

Philip Holland

Jacinta Holmes

Natasha E. Holmes

Nusrat Homaira

Jin-Liern Hong

Dongsheng Hong

Ruoxi Hong

Jiaxu Hong

Il Ki Hong

Sung Kyu Hong

Alejandro Horga

Karoline Horisberger

Takahiko Horiuchi

Tomasz Horoch

Peleg Horowitz

Melanie Horter

Satoshi Hoshide

Qingtao Hou

Juliet Hou

Ming-Chih Hou

James Howard

Reuben Howden

Robert Hoyt

Irena Hrstić

Shao-Hsuan Hsia

John Hsiang

Ming-Yen Hsiao

Yi-Hsien Hsieh

Hui-Shan Hsieh

James Hsieh

Yi-Chen Hsieh

Li-Jen Hsin

I-Fang Hsin

Ching-Sheng Hsu

Chia-Yang Hsu

Chih-Hsin Hsu

Jui-Ting Hsu

Pokuei Hsu

Chao-Wei Hsu

S.-J. Hsu

Yue Hu

Chengbin Hu

Xiaoyu Hu

Xiang Hu

Jiong Hu

Chaur-Jong Hu

Xiaoming Hu

Jian-Kun Hu

Yu Hu

William Hu

Yunwen Hu

Yujian Huang

Hui Huang

Lu Huang

Chihyuan Huang

Yue Huang

Chien-Cheng Huang

Jee-Fu Huang

Cheng Huang

Chao Huang

Chenfei Huang

Kuo-Chin Huang

Li-Kai Huang

Chi-Ling Huang

Chun-Che Huang

Shih-Ting Huang

Yan Huang

Suming Huang

Po-Yen Huang

Yi-Hsiang Huang

Yu-Chuen Huang

Chin-Chou Huang

Hefeng Huang

Gangxiong Huang

Guofu Huang

I-Fei Huang

Shengping Huang

Xiangwei Huang

Wen-Hung Huang

Yu-Yao Huang

Lowiek Hubers

Martin Hubner

Shih-Han Hung

Li-Wei Hung

Chi-Chih Hung

Yao-Min Hung

Chao-Hung Hung

Kuo-Chun Hung

Oliver Hunsicker

Yongliang Huo

Okasha Hussein

Gregor Hutter

Olivier Huttin

Kim Hieu Huynh

Chung-Feng Hwang

Emily Hyle

Noreen Hynes

Jong Jin Hyun

Gianluca Iacobellis

Maurizio Iacobone

Claudio Iaconetti

Carlos Ibañez

Tomoaki Ichikawa

Ancuta Ignat

Katsunori Iijima

Haku Iizuka

Kei Ikeda

Kristian Ikenberg

Baris Ikitimur

Ahmet Ömer Ikiz

Mustafa Ilik

Marie-Christine Iliou

Kenichiro Imai

Zaid Imam

Sara Imanpour

Irfan Inan

Bilsev Ince

Ciro Indolfi

Satoshi Inoue

Pablo Irimia

Vivian Isaac

Alex Isaacs

Hiroyuki Isayama

Lukas Iselin

Shizukiyo Ishikawa

Junichiro Ishioka

Mitsuaki Isobe

Naim Issa

Ahmet Issin

Tomislav Ivanković

Yoshitsugu Iwakura

M J Jacob

Korula Jacob

Tohid Jafari Koshki

Ferenc Jakab

Sridhar Jaligama

Muhsin Jamal

Prince James

Benjamin James

Roland Dominic Jamora

Ewa Janczewska

Byoung Kuk Jang

Insoo Jang

Angelika Janitzky

Koen Janssen

Ed Jarroll

R Jasmin

Nicolas Javaud

Adil Javed

A Jawhar

Anamarija Jazbec

Kevin Jean

Anis Jellad

P Jemenez-Fonseca

Helen Jenkins

Chang-Chyi Jenq

Jin Pyeong Jeon

Eric Jeziorski

Rajneesh Jha

Tuo Ji

Gong-Jun Ji

Dar-Der Ji

Wu Ji

Guiyuan Ji

Yi Ji

Linong Ji

Zhigang Ji

Rui-Peng Jia

Long Jiang

Yusheng Jiang

Weijian Jiang

Yizhou Jiang

Wei Jiang

Li Jiang

Xuejuan Jiang

Shuai Jiang

Xinyi Jiang

Dekejiang Jiang

Qiang Jie

Liu Jie

Patricia Jimenez

Rodrigo Jiménez-García

Félix Javier Jiménez-Jiménez

Lin Jin

Junfei Jin

Wen Jin

Song Jin

Jun Jin

Atul Jindal

Masahiro Jinzaki

Young Suk Jo

Christer Johansson

Angela Johnson

Jeffrey A Jones

Erika Jones

Gwo-Ping Jong

Dong Jin Joo

Heesoo Joo

Kirk Jordan

Alberto Agustín Jorge-Mora

Eric Jorgenson

Prathamesh Joshi

Narendra Joshi

Ankur Joshi

R. San Juan

Javier Juega-Mariño

Giada Juli

Sandeep Julka

Deven Juneja

Ioan Jung

Kyu Sik Jung

Nakajima K

Mark Kaddumukasa

Andre Kaelin

Alisan Kahraman

Ulrike Kaiser

Theodoros Kalampokas

Emmanouil Kalampokas

Hayrapet Kalashyan

Gopal Kaliappan

Ashwini Kalshetty

Umut Kalyoncu

Muhammad Kamal

Avinash Kambadakone

Arnoud Kamman

Konstantinos Kamposioras

Andrew Kan

Rajashekhar Kanchanapally

Raquel Kanda

Eleanor Kane

Laiyi Kang

Hyeun-Ah Kang

Seok Hui Kang

Jung Hun Kang

Wonseok Kang

Meganathan Kannan

Petr Kanovsky

Reba Kanungo

Chih-Chin Kao

Hung-Wen Kao

Dheeraj Kapoor

Eftychia Kapsalaki

Adnan Karaibrahimoğlu

Pantelis Karaiskos

Julia Karakoumis

Timokratis Karamitros

Yaşar Karataş

Samuel Käser

Eiji Kashiwagi

Ronnie Kasirye

Seyed Ebrahim Kassaian

Vilena Kašuba

Gohei Kato

Gagandeep Kaur M.D

Shivani Kaushik

Fotini Kavvoura

Takehiko Kawaguchi

Takashi Kawahara

Mitsuhiro Kawano

Kousaku Kawashima

Cemal Kazimoglu

Saito Kazuyoshi

Colm Keane

Ayse Kefeli

Lindsay Keir

Péter Bertalan Kelemen

Mustafa Keles

James Kellam

Sergio Kellesarian

Jane Kellett

Clive Kelly

Carl Kendall

Amy Kennedy

Justin Keogh

Jesper Kers

A. Kersten

Ahmet Keskin

Satish Khadilkar

Salama Khaled

George M Khalil

Mohamed Khalil

Azadeh Khalili

Humaira Khan

Mohammad Aslam Khan

Muhammad Ali Khan

Sadia Khan

Ashish Khandelwal

Kanika Khandelwal

Elham Kharazmi

Kay-Tee Khaw

Rohan Khera

Sepideh Khodaverdi

Chiea Chuen Khor

Rahul Khosla

Kareeann Khow

Alkesh Khurana

Seda Kibaroğlu

Veysel Kidir

Ursula Kiechl-Kohlendorfer

Thomas Kienbacher

Selim Kılıç

Ibrahim Kılınc

Young-Kug Kim

Dong-Jun Kim

Rock Bum Kim

Young-Hak Kim

Jaihwan Kim

Tae-Hun Kim

Yong Kyun Kim

Nam-Kyu Kim

Tae Min Kim

Jong Hae Kim

Mi Na Kim

Dong Ki Kim

Kun Hyung Kim

Ywan Kim

Byung-Su Kim

Donghee Kim

Chang Woo Kim

Jong-In Kim

Hyungjoon Kim

Deok Won Kim

Michelle Kim

Min Jung Kim

Hyoun-Ah Kim

Eun Soo Kim

Dae Jung Kim

Beom Kyung Kim

Yae Jean Kim

Young Hoon Kim

Sunjung Kim

So Ri Kim

Karen King

Eric Kipnis

Sibel Kiran

Matthew Kircher

Simon Kircher

Eric Kirschner

Marcio Kiuchi

Sverre Kjeldsen

Nadja Klafke

Janssen Klaus-Peter

Jorg Kleeff

Rainer Klocke

Petra Klose

Jens Klotsche

Roger Klotz

Jeffrey Knauf

Von Dem Knesebeck

Mladen Knotek

Eva Cecilie Knudsen

Gyung Hyuck Ko

Rakesh Kochhar

Bérengère Koehl

Ingo Koenigs

Willibald Kohlhepp

Judith Kohlmeyer

Javad Kojuri

Victor Kok

Alexander Kokkinos

Matthieu Komorowski

Muralidhar Kondapaneni

Hiroshi Kondo

Elissaios Kontis

Jaehyung Koo

Jennifer Koplin

Andrei Koptioug

Panagiotis Korovessis

Stephen Korsman

Farhad Kosari

Isabelle Koscinski

Michael S. Kostapanos

Shigeru Kotake

Magd Kotb

Vishal Kothari

Jignesh Kothari

Amol Kothekar

Vikram Kotwal

George Koukourakis

Dimitrios Koutoukidis

Mitat Koz

Piotr Kozlowski

Diomidis Kozyrakis

Tanja Kraus

K Krause

Matthias Kreppel

Fabian Kriesel

Lakshmi Krishnakumar

Sharmitha Krishnamurthy

Venkatesh Krishnan

Jayalakshmi Krishnan

Peter Kruzliak

Taishi Kswahara

Nam Su Ku

Yi-Chun Kuan

Anren Kuang

Toshifumi Kudo

Gerhardus Kuiper

Michał Kukla

Ravina Kullar

Virendra Kumar

Manoj Kumar

Dushyant Kumar

Sandeep Kumar

Nitasha Kumar

Abhishek Kumar

Shaji Kumar

Dilip Kumar

Sanjeev Kumar

Chang-Fu Kuo

Te-Hui Kuo

Galip Bilen Kürklü

Vladimir Kushnir

Semanur Kuyucu

Yong Hwan Kwon

Seong Young Kwon

Yok Lam Kwong

Naylor Kyla

Jessica Kynyk

R La Joie

Ismail Labgaa

Alexandre Laedermann

Tim Lahm

Li Lai

Eric Ch Lai

Jung-Nien Lai

Ming-Wei Lai

Chih Hung Lai

Vaia Lambadiari

Fiona Lampe

Dadja E Landoh

Marieke L.A. Landsmeer

Sven Lang

Thomas Langenickel

Cord Langner

Fanny Lanternier

Constantin Lapa

Jan Larmann

Joseph Larmarange

Romy Lauche

Camile Laurent

Carl Lavie

Giagkos Lavranos

Liora Lazar

Reynaldo Lazaro

Hillard Lazarus

Chiara Lazzeri

Kang Le

Florence Le Calvez-Kelm

Florent Le Ven

Pamela Leal

Karin Leander

Jeannette Lechner-Scott

Chih-Hsin Lee

Dong Soo Lee

Haekyung Lee

Gun Woo Lee

Jeong-Hoon Lee

Changhyun Lee

Jongmin Lee

Jae Gil Lee

Jung Hwan Lee

Li-Ang Lee

Seung-Hwan Lee

Kyong Joo Lee

Ying-Hsiang Lee

Yi Che Lee

Yong-Ho Lee

Yuan-Chieh Lee

Cheng-Hung Lee

A Leum Lee

Hyangsook Lee

Ho-Won Lee

Hye Won Lee

Kuei-Chuan Lee

I-Cheng Lee

Hye Ah Lee

Yong Chul Lee

Sukmin Lee

Meng Lee

Malrey Lee

Sae Hwan Lee

Dong Ho Lee

Dong Ki Lee

Myeong Soo Lee

Jen-Kuang Lee

Mel S. Lee

Chien-Chang Lee

Yu-Chien Lee

Su Jin Lee

Jérémie Lefèvre

Stephane Legriel

Wenbin Lei

Rogelio Leira

Roman Leischik

Andrea Lembo

Cédric Lemogne

Maud Lemoine

Jose Lemos

Manuel Lemos

Malcolm Lemyze

Jacques Lenders

Cédric Lenormand

Frank Lenze

Marc Leone

Sebastiano Leone

Grigorios Leontiadis

Giuseppe Lepore

Emmanuel Letavernier

Claudio Letizia

Sonja Levanat

Nicolas Leveziel

Cara L Lewis

Martin Lhuaire

Sheyu Li

Hui Li

Gang Li

Xingyao Li

Chien-Feng Li

Yingxin Li

Liming Li

Bin Li

Hua Li

Yaning Li

Wenzhao Li

Quanlin Li

Qiang Li

Zhentian Li

Jie Li

Zhaoyu Li

Wenting Li

Ruifang Li

Shiri Li

Yuhuang Li

Zhuyun Li

Lieber Po-Hung Li

Jingting Li

Dan Li

Ningdong Li

Yifei Li

Hecheng Li

Xi Li

Yuping Li

Yan Li

Xiaomin Li

Dali Li

Zhi-Jun Li

Jinrong Li

Yijia Li

Ning Li

Jun Li

Jingmei Li

Yu-Chuan (Jack) Li

Guoqiang Li

Hong Li

Xiaoni Li

Yi Li

Shi-Jun Li

Yang Li

Changzheng Li

Mengtao Li

Ming Li

Emmy Li

Guojun Li

Guoan Li

Chung-Pin Li

Lin Li

Sheng Li

Baosheng Li

Liliang Li

Jiangfa Li

Chia-Hsiang Li

Yuqing Li

Xiaoshan Li

Xiao-Feng Lian

Kaiwei Liang

Chen Liang

Zhipin Liang

Wenjie Liang

Houjie Liang

Deguang Liang

Wang Lianghua

Wenjun Liao

Kuang-Ming Liao

Chien-Hung Liao

Lingjie Liao

Wei Liao

Chun-Ta Liao

Chien-Wei Liao

Pen-Chih Liao

Christoff Liebetrau

Rogozea Liliana

Tze Peng Lim

Renly Lim

Luiz Lima

Cláudia Lima

Shuibin Lin

Tonghui Lin

Hugo Lin

Yi-Zhi Lin

Ruiting Lin

Chuwen Lin

Ming Kai Lin

Chun-Ju Lin

Lian-Yu Lin

Pyng-Jing Lin

Yuan Lin

Cheng-Jui Lin

Che-Hsuan Lin

Lifeng Lin

Jingmei Lin

Lizhou Lin

William Lin

Yansong Lin

Frank Lin

Jie Lin

Cheng-Wen Lin

Chia-Hung Lin

Jui Hsiang Lin

Sheng-Hsiang Lin

Yu-Pang Lin

Joanne Lind

Yun Ling

Hua Ling

Dai Lingzhen

Michael Link

D Lipsker

Rose Lisboa

Ou Liu

Xing Liu

Chun-Hua Liu

Jie Liu

Jian Liu

Xuhang Liu

Xu Liu

Tao Liu

Ruya Liu

Ying Liu

Lei Liu

Sai Liu

H Liu

Jinghua Liu

Huayang Liu

Jinxu Liu

Ching-Feng Liu

Yi Liu

Meng Liu

Yijie Liu

Shijie Liu

Yuxuan Liu

Junjie Liu

Jianping Liu

Kaiyong Liu

Chuanxu Liu

Chung-Pin Liu

Miao Liu

Feng Liu

Ziying Liu

Wenzhong Liu

Rengyun Liu

Chao Liu

Peng Liu

Xin Liu

Cheng Liu

Kai-Xiong Liu

Hua Liu

Haizhou Liu

Zhao-Min Liu

Yong Liu

Shih-Feng Liu

Qi-Fa Liu

Sijun Liu

Tong Liu

Lijun Liu

Yanqiong Liu

Min Liu

Michael Liu

Nan Liu

Chun-Yu Liu

Yu Liu

Yan Liu

Raymond Liu

Jorge Lizandra

Josep M Llibre

Bo Løfgren

Mark Logue

Carlo Lomonte

Zaiyang Long

Jun Long

Lingyun Long

Xiao Long

Samantha Longman-Mills

Benjamin Longo-Mbenza

Bert Lopansri

Luis Lopes

Rafael López-Andújar

Alejandro López-Magallon

Gunnar Loske

Francesco Lotti

Grant Louie

Carlos Lozoya-Ibáñez

Yunqi Lu

Zhenwei Lu

Rongze Lu

Chieh-Sheng Lu

Yi-Fan Lu

Zuxun Lu

Meixia Lu

Yuan-Qiang Lu

Chia-Wen Lu

Chang-Hsien Lu

Lu Lu

Xuyang Lu

Bin Lu

Ying Lu

Giuseppe Lucarelli

Yvonne Lucero

Randy Luciano

Rafael Luciano

Lamb Lucy

Jason Luke

Livia Lumbroso-Le Rouic

Carlotta Lunghi

Wenting Luo

Biquan Luo

You Luo

Rong Luo

Xiaohe Luo

Ying Luo

Vasile Valeriu Lupu

Jay Luther

Weiran Lv

Zhibao Lv

Anastasios Lymperopoulos

Theodore Lytras

Xiaoxuan Lyu

Marcia M.M. Pizzichini

Bo Ma

Tianle Ma

Yan Ma

Qianyi Ma

Chiyuan Ma

Jun Ma

Chong Ma

Ismaël Maatouk

José Fernando Macedo

Fabiana Machado

Rh Mackey

Clelia Madeddu

Heloísa Maestrini

Leon Maggiori

Dianna Magliano

Pj Maglione

Rohan Mahabaleshwarkar

Surakameth Mahasirimongkol

Susan Malcolm Smith

Benedict Maliakkal

Pavlos Malindretos

Vijetha Maller

Murali Malligaraman

Basant Malpani

Andrew Mammen

Caterina Mammina

Nodoka Manabe

Mattias Mandorfer

Charalampos Mandros

Fabio Mangiacapra

Chinnadurai Mani

Ramakrishnan Mani

Nandini Mani

Elias Manjarrez

Rebecca Manno

Palanikumar Manoharan

Spilios Manolakopoulos

Sobia Manzoor

Mao Mao

Qianguo Mao

Chen Mao

Jianhua Mao

Saw Ohn Mar

Dario Marangoni

Luigi Marano

Dror Marchaim

Adil Mardinoglu

Giancarlo Marenzi

Masarone Mario

Daniel Markel

Charlotte Markert

Daniele Marrelli

Aldo Marrone

Vito Martella

Sabina Martinenghi

Esteban Martinez

Magdalena Martinez-Tome

Fernando Martínez-Valle

Wellington Martins

Yasufumi Masaki

Ferran Masanes

Dipak Maskey

Sara Massironi

Satohiro Masuda

Takayuki Masui

A Matsuada

Kazumasa Matsumoto

Hisako Matsumoto

Nobuaki Matsunaga

Clio Mavragani

Christos Mavrommatis

M Mavros

Austin J May

Teresa Mayordomo-Rodriguez

Johannes Mayr

Toshihiko Mayumi

Daniel Ferraz De Campos Mazo

P M’Bappé

Devin Mccaslin

Brian Mcclune

Rozalina G. Mccoy

Colleen Mcdowell

Jennifer A. Mcgowan

Bradford Mcgwire

Craig Mclachlan

J. Chase Mcneil

Stephen Mcsorley

Luis Medina Andrade

Reinier Meester

Bruno Megarbane

Christine Mehner

Cláudia Meirelles

Mohamed Mekky

Lorena Elena Melit

Yongfan Men

Fabrice Menegaux

Zhipeng Meng

Zhaowei Meng

Weixu Meng

Zhefeng Meng

Chengbo Meng

Cong Meng

Linghui Meng

Jie Meng

Vidya Menon

Vipin Menon

Lucile Mercadal

Shaila Merchant

Ryan Merrell

Neil Merrett

Tamã¡s Mersich

Thibault Mesplede

Claudio Mesquita

Fabiana Mesquita Barros

Ramy Messiah

Alain Meyer

Jeffrey Meyer

Tammy Meyers

D Meyre

Silvia Miano

Yuxuan Miao

Antonios Michalopoulos

Josef Michl

Sofie Midgley

Diego Milani

Mirta Milić

Cameron Millar

Eli M Miloslavsky

Deng Min

Hitomi Minami

Janos Minarovits

Xie Ming

Gesthimani Mintziori

Fabio Minutoli

Adnan Mir

Jose M Miro

Silvia Misasi

Subhasis Misra

Benjamin Misselwitz

David Mitchell

Deepanjan Mitra

Vinay Mittal

Takashi Miura

Masaru Miura

Kentaro Miyai

Xiulei Mo

Aaron Moberly

Simone Mocellin

Manabu Mochizuki

Eduardo Moffa

Parul Mohan

Rajesh Mohandas

Mina Mohareb

Markus Mohaupt

Sydney Moirangthem

Xavier Moisset

Ikhsan Mokoagow

Javier Molina-García

Mariana Monteiro

Martin Montenovo

Luke Moore

William Moore

José Morales

Carolina Moreira

Gustavo Moreira

Alejandro Moreno

Rayo Morfin-Otero

Howard Morris

Brian Morris

Majid Moshirfar

Timothy Moss

Norhan Mostafa

Taymour Mostafa

Shinichiro Motohashi

Seyed Mousavi

Cyril Mousseaux

Amirhossein Mozafarykhamseh

Olga Mozenska

Yunxiang Mu

Liangshan Mu

Muhammed Mubarak

Siegfried Muhlack

Nabanita Mukherjee

Tanushri Mukherjee

Irene Mukui

Giuseppe Mulè

Xavier Muller

Sebastian Müller

Andreas Müller

Philip Müller

Beat Müller-Stich

Razvan Multescu

Olavo Munhoz Leite

Agustin Muñoz Sanz

Pelagia Murangandi

Andrew Murphy

Earnest Murray

Dedee Murrell

Taisei Mushiroda

Maria Carolina Mussi

Pellegrino Musto

Nair Muto

Jonathan Myers

Manikandan N

Iraj Nabipour

Paweł Nachulewicz

Ramos Nacimiento

Rajakumar Nagarajan

Yoji Nagashima

Ricardo Nahas

Bhiken Naik

Suresh Nair

Narendra Nair

Ahed Najjar

Abul K. Najmi

Rine Nakanishi

Haruki Nakano

Michihiro Nakayama

Farid Nakhoul

Vinod Nambudiri

Mehdi Namdar

John Nance

Santosh Nandigam

Sadhna Nandwana

Kanwar Narain

Nandakumar Narayanan

Fabio Nascimbeni

Androniki Naska

Fabio Natale

L. Gabriel Navar

Yoshio Naya

Chidozie Nduka

Elizabeth Neale

Alan Needle

Kari Neemann

Richard Nelson

Alexandr Nemec

Binod Nepal

Piergiorgio Neri

Christopher Nettles

Arlene Ng

Curtise K.C. Ng

Alex Lk Ng

Qingqiang Ni

Cluas Niederau

Antoni Niedzielski

Patricia Nielsen

Ole Haagen Nielsen

Henrik Nienhüser

Torbjörn Nilsson

Peter M Nilsson

Zhen Ning

Grazia Niro

Hiroshi Nishida

Tsutomu Nishiyama

Ulrich Nitsche

Jun Niu

Nicolas Noel

Osamu Nomura

John Paul Norvell

Alexander Novotny

Maja Nowakowski

Anna Nowinska

Saad Nseir

Dimitrios Ntourakis

Alex Nunes

Rodolfo Nuñez-Miller

Michael Nurmohamed

Eric Nylen

Joo Hyun O

Stephen Obaro

Christian Oberkofler

Paulo Ocke Reis

Brendan O’Connell

Ichiro Oda

Ryo Oda

Mesut Ogrendik

Young-Min Oh

Steven P. O’Hara

Masato Ohsawa

Yuichi Oike

A Oishi

Norma Ojeda

Tomohisa Okada

Takahiro Okamoto

Manabu Okawada

Ikechi Okpechi

Yoichiro Okubo

Minoru Okubo

Jose Mario Franco Oliveira

Priscila Oliveira

Luis Vicente Franco Oliveira

Gustavo Oliveira

Mauro Oliveira

Joao Paulo Oliveira-Costa

Joanne Oliver

Rosemary Olivero

Karina Olsen

Masami Omae

Philip Omotosho

Go Omura

Gokalp Oner

Veysi Öner

Catherine Ong

Franz Onishi

Suzane Ono

Jot Hui Ooi

Ana-Maria Orbai

Lorenzo Orci

Carlos M. Ordás

Janina Orlowska

Laura Orsolini

Hisashi Oshiro

Alexi Otrakji

Gordon Otto

Abderrahim Oussalah

Edgar Turner Overton

Genko Oyama

Bilgehan Oz

Birsin Ozcakar

Orhan Özdiler

Kemal Emre Özen

Y Ozguler

A Ozyilmaz

Elisabetta Pace

Jose Pacheco-Nunez

Matteo Pacini

Mahesh S Padanad

Atul Padole

Elvira Padua

Johnny Padulo

Michael Paech

Jin Chul Paeng

Alessio Paffoni

Spyridon Pagkratis

Carlos Paiva

Antonio Palazón-Bru

Nicola Palestini

Narayan Palla

Carolina Palmela

Manisha Palta

G Palumbo

Orestis Panagiotou

Rohit Panchakshari

Arjun Pandey

Nandini Pandit

Hua Pang

Vincenzo Panichi

Chris Panopoulos

Chaitanya Pant

Nikos Pantazis

Simon Panter

Satyabrata Pany

Francesco Panza

Antonio Paoli

Antonis Papadopoulos

Chryssanthi Papadopoulou

Konstantinos Papamichail

Stefania Papatheodorou

Dominic Paquin Proulx

Balaji Parameswaran

Dimitrios Paraskevis

Juan A. Pareja

Parag Parekh

Rahul Parghane

S Parida

Anit Parihar

Kathan Parikh

Purvi Parikh

Samir Parikh

Wooyeong Park

Chanhyun Park

Byung-Joo Park

Kwan Park

Ha Na Park

Su-Hyung Park

Shefton Parker

Barbara Parolini

Olavi Parssinen

Iffat Parveen

Ahmed Pasha

Massimo Pasquini

Francesco Passafaro

Clévia Dos Santos Passos

Rogerio Passos

Ives Passos

Jacyr Pasternak

Girijesh Patel

Darshan Patel

Bhupeshwari Patel

Breay Paty

Massimililano Pau

Shwetal Pawar

A Cemal Pazarli

Oana Pecurariu

Eva, Pedersen

Corrado Pedrazzani

Enric Pedrol

Paula Peixe

Ronald Pelletier

Hui Peng

Cheng-Yuan Peng

Nilson Penha-Silva

Carl Pepine

Camille Perchoux

Valter Luis Pereira Jr.

María Jesús Pérez Elías

Santiago Perez Patrigeon

Miguel Pérez-Fontán

Kostas Perisinakis

Fabienne Perren

Carlo Perricone

Francesco Perticone

Milica Pesic

Katherine Peters

Milena Petranovic

Fausto Petrelli

Andrew Petroll

Mladen Petrovečki

Carmel Pezaro

Raffaele Pezzilli

Thai Pham

Femi Philip

Andrea Picchianti-Diamanti

Francesco Pichi

David Pieper

Guillaume Piessen

Fernanda Pigatti

Roberta Pili

Trinadha Pilla

Micheli Pillat

Karin Pillunat

Luiz Pimenta

Matthew Pincus

Marco Pinho

Basilio Pintaudi

Ignazio Piras

Claudia Pisanu

Alica Pizent

Ioannis Pliakos

Mauro Podda

Patricio Polanco

Alberto Polimeni

Stuart Polisner

Sean Polster

Angelo Pompucci

Antonio Ponzetto

Dimitri Popkov

Silvia Porcu

Goran Poropat

Jesús Porta-Etessam

Renata Pospischill

F Post

Nicolas Poté

Evangelos Potolidis

Julien Pottecher

Harish Potu

Mathias Poussel

Ana Póvoa

Vincent Pradel

Marina Prado

Vicente Prado-Gasco

Gaurav Prakash

Marie Preau

Fabio Presotto

Melissa Price

Jose Ignacio Priego Quesada

Belen Prieto

Sergio Prieto-González

Roberta Priori

Pascal Probst

Giuseppe Procopio

Matthew Prof. Cook

Patrizia Proia

Veronique Provencher

Mina Psichogiou

Kuba Ptaszkowski

Jun Pu

Giacomo Pucci

Gonzalo Pullas

Raffaele Pulli

Ameya Puranik

G.D. Puri

Vincenzo Puro

Yuchen Qi

Dongfei Qi

Qibin Qi

Xiaoqiang Qi

Zhen Qi

Yuan-Yuan Qi

Xi Qian

Xu Qian

Pengxu Qian

Aijun Qiao

Shuxi Qiao

Lili Qin

Gang Qin

Zhengtao Qin

Fujun Qin

Wei Qin

Ruofeng Qiu

Zhi-Bing Qiu

Yun Qiu

Yudong Qiu

Yong Qiu

Jinfeng Qu

Zhe Qu

Luciano Quaranta

Diego Quattrone

Paola Queirolo

Angela Quirino

Abdul Qureshi

Brandon Rabeler

Ann Raes

Mahmoud Rafieian-Kopaei

Ralph Rahme

M G R Rajan

Anna Ralph

Meenakshi Ramanathan

Arvind Ramanujam

Albert Adrien Ramelet

Paula Ramírez

Jm Ramos

Surinder Rana

Dharshan Rangaswamy

Rajni Rani

Jonas Ranstam

Tuomo Rantanen

Sheng-Xiang Rao

Bhaskar Rao

Sunil V Rao

Jean-Luc Raoul

Shah Manzur Rashed

Armin Rashidi

Rajah Rasiah

Mustafa Rasid Toksoz

Retty Ratnawati

Luis Rato

Uffe Ravnskov

Dhwaj Bahadur Rawat

Pratima Rawat

Steve Rayhill

Stéphanie Raymond

Christian Récher

Uttam Reddy

Donald Redelmeier

Ravinder Regatte

Alfonso Reginelli

Rebecca Reid

Daniel Reim

Stephen Reis

Jochen Reiser

Hong-Gang Ren

Pingping Ren

H. Ren

Jianan Ren

Georgios Rentzos

Cristina Reschke

Soledad Retamozo

Stefan Reuter

Yousef Rezaei

Daniel Ribeiro

Salma Ribeiz

Josep-Maria Ribera

Simone Ribero

Corey Richardson

Stefano Rigattieri

Diego Ripamonti

Frédéric Ris

Martin Risch

Shemra Rizzo

Gregory Robbins

Victor Roberts

Matthew Roberts

Ricardo Robles

Manoel Rocha

Luis Rodrigo

Rodrigo Rodrigues

Alexander Rodriguez

Daniel Rodríguez

M Rodriguez Esteban

Rene Rodriguez Gutierrez

B Rodríguez-Alfonso

Gregorio Rodríguez-Boto

William Rodríguez-Cintrón

Mayela Rodriguez-Violante

Franz Roedel

Christopher Roman

Rizwan Romee

Ricardo Romiti

Giuseppe Romito

Nicoletta Ronda

Xiaoxiang Rong

Annerine Roos

Raymond Rosales

Alexander Rösch

Christopher Rose

Meng Rosie

Yossi Rosman

Sandor Roth

Christos Rountas

Marie Rousset

Debasish Roy

Cathy Rozmus

João Ruaro

Kurt Ruetzler

Andrés Eduardo Ruf

Maria Ruffino

Loredana Ruggeri

Javier Ruiz-Martinez

Jody A Rule

Christian Rupp

Daniel Rushing

Maurizio Russello

Antonio Russo

Andrea Ruzzenente

Jenna Rychert

Ji Kon Ryu

Abdelilah S. Gounni

Rosália Sa

Khaled Saad

Akmal Sabarudin

João Sabino

Maja Sabol

Francesco Saccà

George Sack

Tsuneaki Sadanaga

Isabel Sada-Ovalle

Recep Sade

Ruxana Sadikot

Ali Reza Safarpour

Oana Säƒndulescu

Junichiro Sageshima

Evangelista Sagnelli

Sunandan Saha

Ruta Sahasrabudhe

Alparslan Sahin

Muhammed Sahin

Alpaslan Sahin

Zafer Şahin

Ilknur Şahin

Gökalp Şahin

Tuncay Sahutoglu

Kentaro Sakamaki

Umut Sakarya

George Sakoulas

Habeeb Salameh

Gerard Salazar

Paul Saleeb

Mohamed Saleh

Nadia Salerno

Alicia Sales-Galan

Ayse Salihoglu

Ozan Salim

Federico Salomone

Roberto Salvatori

Paolo Salvi

Roberto Salvia

Alexandros Samartzis

Julia Samuelson

Ravi Samy

María Esther Sánchez-García

Philipp Sand

Alison Sanders

Jose Sandoval

Jhuma Sankar

Saikolappan Sankaralingam

Sethuraman Sankaran

Vishwanath Sankarasubramanian

Catarina Santos

Rita Santos Rocha

John Santoshi

Sonia Santos-Lasaosa

Gaetano Santulli

Janio Santurio

Neeraj Saraf

Bruno Saragiotto

Claudia Sardu

Anggraini Sari

Shiv Kumar Sarin

Rajiv Sarkar

Rani Sarkis

Manoj Sarma

Smitha Sasindran

Pamela Sass

Bulent Satar

Mike Sathekge

Michihiro Satoh

Osawa Satoshi

Bipin Savani

Edoardo Savarino

Emilie Sbidian

Esperance Schaefer

Bernhard Schaller

Jörn Schattenberg

Peter Schemmer

Paolo Schena

Stefan Schenk

Jürgen Scherberich

Oliver Schildgen

Lisa Schimmenti

Christoph Schliemann

Christoph Schmaderer

Sarah Schmalzle

André Schmidt

Dieter Schmidt

Roland Schmieder

Jochen Schneider

Markus Schneider

Simon Schneider

Hans Schoellhammer

Michael Scholl

Wenzel Schöning

Benjamin Schreiber

Dania Schumann

Steven Schwartz

Robert Scofield

Nicholas Sculthorpe

Antonio Secchi

Bruno Sedassari

Anil Seetharam

Giuseppe Seghieri

Nereo Segnan

Ana Catharina Seixas Santos

Jacobo Sellarés

Ishita Sen

Shaundeep Sen

Eric Senneville

Michele Senni

Mehmet Şentürk

Yeon Seok Seo

Philip Seo

Sadaf Sepanlou

Masood Sepehrimanesh

Patrick Sequeira-Byron

Anna Serafini

Sancar Serbest

Giovanni Serio

Juan Serna

Carmen Serna-Candel

Pilar Serra-Añó

Filiberto Serraino

Gil Serrancoli

Barış Sevinç

Guzin Sevincer

Mathew Sewell

Ulrich Seybold

Adonis Sfera

Tariq Shafi

Priyank Shah

Zulfin Shaikh

Guogen Shan

Satish Shanbhag

Prapimporn Shantavasinkul

Di Shao

Sida Shao

Shi Shao Hua

Ron Shapiro

Deepak Sharan

Surendra Sharma

Vishal Sharma

Rohini Sharma

Suvasini Sharma

Tai Mei Ling Sharon

Bronwen Shaw

Qiang She

Sameh Shehata

Yanguang Shen

Hsiu-Nien Shen

Jiangang Shen

Yinzhong Shen

Feng Shen

Jianzhen Shen

Jie Shen

Tao Shen

Shaomin Shen

Jin Sheng

Laila Sherief

Morris Sherman

Sameer A. Sheth

Chau-Chyun Sheu

Yingtang Shi

Bingyin Shi

Xiaoshun Shi

Jun Shi

Mei Shi

Tzong-Ming Shieh

Jin-Chung Shih

Kendrick Shih

Byung-Cheul Shin

Hyun-Woo Shin

Dong Yeong Shin

Toshiharu Shirai

Anushree Shirali

David Shitrit

R Shivaprakash

Yehuda Shoenfeld

Shmuel Shoham

Peishun Shou

Xiang Shu

Sudhanshu Shukla

Jennifer Shum

Jay Siak

Ahmed Siam

Rosa Sicari

Rinaldo Siciliano

Dr Cristina Sierra

Walter Sifuentes-Giraldo

Patima Silsupadol

Antônio Eduardo Silva

Matthew Silva

Brian Silver

Amanda Simanek

Olga Simionescu

Michael Simon

Miroslav Simunic

Marc Singer

Sanjay Singh

Soudamani Singh

Kern Singh

Anup Singh

Sunita Singh

Jaspal Singh

Baljinder Singh

Brajesh Singh

Aditi Sinha

Dong Hyun Sinn

Phumla Sinxadi

Csilla Sipeky

Mrinal Sircar

Ajith Siriwardena

Banu Sis

Folke Sjoberg

Stamatios Skevofilax

Nina Skjæret Maroni

Riemer Slart

Vina Slev

Artur Slomka

Julie Smith Gagen

Kim Smith-Whitley

Chelvin Sng

Christopher So

Ja Sokol

Naimesh Solanki

Marco Solmi

Hirohito Sone

Qingxuan Song

Yan Song

Yoo Sung Song

Peipei Song

Sheng Song

Mei Song

Jeong Eun Song

Kapil Dev Soni

Mehmet Sonmez

Tomohiro Sonoo

Ashwani Sood

Alessandro Soria

Yasunori Sorimachi

Sabato Sorrentino

Enrique Soto Pedre

Mirian Sotto

Hugo Soudeyns

Tayana Soukup

Eric K Soule

Suzana Sousa

Michael Soussan

Leonard Sowah

Mehmet Enes Sözen

Mónica Sparo

Logan Spector

Robert Speth

Loukia Maria Spineli

Bruno Spire

Jason Springer

Nicola Squillace

Jeffrey Staab

Richard Stabler

Anamarija Štafa

Tijana Stankovic

Catherine Staton

Katharina Staufer

Philipp Stawowy

Nico Steckhan

Sonja Steigen

Felix Steiger

Robert Steiner

Dominik Steubl

Timothy Stockwell

Mark Stokes

Katarzyna Stolarz-Skrzypek

Lahn Straney

Anca Streinu-Cercel

Pasquale Striano

Oliver Strobel

Liborio Stuppia

Yan Su

Qiang Su

Shaoyong Su

Lin-Hui Su

Jing Su

Chin-Hui Su

Sui-Lung Su

Chien-Wei Su

Edu Suarez-Martinez

Ramu Sudhagoni

Yuh-Mou Sue

Yasuhiko Sugawara

Haruhiko Sugimura

Jin-Suck Suh

Mina Suh

Shawgi Sukumaran

Abdul Razak Sulaiman

Suman Suman

Zhonghua Sun

Yonghu Sun

Ye Sun

Ang Sun

Yinghao Sun

Yuqing Sun

Tao Sun

Zhiqi Sun

Zhongren Sun

Zhao Sun

Weiping Sun

Celi Sun

Xiaojun Sun

Leimin Sun

Beicheng Sun

Qun Sun

Sumati Sundaraiya

Lone Sunde

Robert Sundel

Cord Sunderkoetter

Fung-Chang Sung

Shih-Hsien Sung

Ki Chul Sung

Li-Chin Sung

Adithan Surendiran

Ashish Suri

Adam Susmarski

Keisuke Suzuki

Shigeaki Suzuki

Kiyoshi Suzuma

Lars Bo Svendsen

Subramanian Swaminathan

Alex Sweeney

Randy Sweis

Peter Sykora

Leszek Szenborn

Krzysztof Szmyt

Leslaw Szydlowski

Balamugesh T

Garden Tabacchi

Azadeh Tabari

Ozra Tabatabaei-Malazy

James Tabibian

Luís Manuel Taborda Barata

Parissa Tabrizian

Sascha Tafelski

Khaled Tafran

Shokouh Taghipour Zahir

Mohammad Taher Tahoori

Ayman Tailakh

Satoru Takahashi

Kei Takayama

Masaru Takeuchi

Hirofumi Taki

Yamada Takumi

Prue Talbot

Kiran Talekar

Nilesh Talele

Reinskje Talhout

Wilson Tam

Domenico Tamburrino

Salem Tamer

Sharon Tamir

Kouichi Tamura

Min Tan

Zhijing Tan

Hua Tan

Maw Pin Tan

Xi Tan

Hong Yien Tan

Xiaojun Tan

Tina Q Tan

Claudio Tana

Hiroshi Tanaka

Yoshiya Tanaka

Kanwarpreet Tandon

Yinghua Tang

Yaohui Tang

Shaojun Tang

Yunneng Tang

Zheng Tang

Danming Tang

Arthur Tang

Jie Tang

Yujie Tang

Wei Tang

Chie Taniguchi

Alpaslan Tanoglu

Tamara Tanos

Herbert Tanowitz

Eda Tanrikulu Simsek

Bekir Tanriover

Wensi Tao

Fang Tao

Huiren Tao

Jie Tao

Luciano Tarantino

Der-Cherng Tarng

Rai Tarun

Marios-Konstantinos Tasoulis

Erhan Tatar

Ichiro Tatsuno

Mimmo Tavella

Gregory Taylor

Maida Taylor

Michael Taylor

Beth Taylor

Caroline Taylor

Alberto Tedeschi

Xm Teitsma

Johanna Temime

Arnoud J Templeton

Miao Teng

Melissa Teo

Boon Wee Teo

Anthony Teoh

Chikashi Terao

Anish Thachangattuthodi

Hamsa Thayele Purayil

Rajesh Thippeshappa

Umapathi Thirugnanam

Ryan Thomas

Astrid Thomas

Dafydd G. Thomas

John Thompson

Erik J. Thoomes,

Rui Tian

Xuebi Tian

Chenxi Tian

Yuan Tian

Xu Tian

Hong-Tao Tie

Yu-Wen Tien

Bedile Irem Tiftikcioglu

G H Tilve

Camilla Tincati

Rasna Tiwari

Giuliano Tocci

Albena Todorova

Daisuke Tokuhara

Maria Tolia

Carmine Tomasetti

Jingshan Tong

Lu-Sha Tong

James Tonsgard

Tayfur Toptas

Michele Torella

Sakuragi Toro

Ayşe Torun

Nobuyuki Toshikuni

Christian Toso

Shigeru Toyoda

Varvara Trachana

Athanasios Tragiannidis

Maxine Tran

Carlo Trani

Marten Trendelenburg

Helen Triantafyllidi

Christos Triantos

Domenico Tricarico

Bruno Trimarco

Priscila Trindade

Kaushlendra Tripathi

Durga Tripathi

Roberto Ivan Troisi

Aleksandra Truszczyńska

Wei-Lun Tsai

Hui-Ji Tsai

Tsungyuan Tsai

Meng-Han Tsai

Susanna Tsang

Ching-Min Tseng

Fen-Yu Tseng

Chih -Wei Tseng

Ing-Jy Tseng

Tai-Chung Tseng

Chi-Chuan Tseng

Konstantinos Tsilidis

Sotirios Tsiodras

Georgios Tsivgoulis

Ioannis Tsougos

Nikolaos Tsoukalas

Fon-Yih Tsuang

Kenji Tsuchida

Huakang Tu

Krishna Tummala

Abdullah Tuncez

Mehmet Turan

Ersin Turan

Sabin Turdean

Fatih Turkcu

Mehmet Turkcu

Kültigin Türkmen

Akif Turna

Massimo Tusconi

Antonino Tuttolomondo

Nikhil Tyagi

Siddhartha Tyagi

Richa Tyagi

Konstantinos Tziomalos

Ag Tzioufas

Ahmet Deniz Uçar

Cemile Ucgul Atilgan

Mehmet Ugurlu

Alexis Ulrich

Engin Ulukaya

Hiroyuki Umegaki

Takeji Umemura

Tevfik Kivilcim Uprak

Muhammet Uraloglu

Thomas Uray

Pablo Uribe

Francesco Ursini

Olalekan Uthman

Tijen Utkan

Omer Uysal

Jose Valbuena

Filippo Valbusa

Luca Valenti

Gabriele Valentini

Gabriel Romualdo Valero

Sreeram Vallabhaneni

Paul Van Daele

Peter Van Der Wurff

Alison Van Dyke

Jan Van Lunzen

Jacobus P Van Wouwe

Vishwas Vanar

Jason Vanatta

Maaike Vancamelbeke

Lisa Vanhouwelingen

Albert Varga

Asha Varghese

Roberta Vasconcelos

Panagiotis Vasilakis

George Vassilopoulos

Janitzia Vazquez-Mellado

Sriram Velandy

Vijayakumar Velu

Sundar Veluswamy

Per Venge

Chamcha Venkateswarlu

Vishwanath Venketaraman

Ramakrishna Venugopalan

Cristian Vera

Chris Verhofstede

Priyanka Verma

Nicola Veronese

Sare Verstockt

Francesca Viazzi

Marco Vieira Guedes

Luca Vigano

Federica Vigna-Taglianti

Ramachandran Vignesh

Sanjiv Vij

Naval Vikram

Ajit Vikram

Mary Vilay

Tomás J Villén

Jean-Louis Vincent

Ashley Vincent

Enzo Maria Vingolo

Francesco Virdis

Efi Vlachaki

Antonios Vlahos

Benoit Voisin

B J Von Scholten

Evangelos Voulgaris

Ivan Vujkovic-Cvijin

Stela Vujosevic

Jason Wachsmann

Adrian Wagg

Martin Wagner

Ingrid Waisman

Ian Wallace

Jeffrey J Walline

Michael Walsh

Zenta Walther

Pengxia Wan

Qiaoqiao Wan

Bin Wang

Weilin Wang

Shan Wang

Yuanyuan Wang

Wei Wang

Kang Wang

Xiaojing Wang

Yanru Wang

Yanqing Wang

Wen-Jun Wang

Rui Wang

Leiming Wang

Xu Wang

Chuanxi Wang

Fu Wang

Manqi Wang

Jann-Tay Wang

Lingling Wang

Xin Wang

Feng Wang

Mengjie Wang

Jianhui Wang

Yucai Wang

Peiji Wang

Quan Wang

Qing Wang

Weiqi Wang

Wenshan Wang

Youbin Wang

Fang Wang

Honglin Wang

Ju Wang

Dan Wang

Liuyang Wang

Gang Wang

Yushan Wang

Shuaishuai Wang

Zhe Wang

Yi-Jun Wang

Changsong Wang

Niansong Wang

Jun Wang

Qiongxin Wang

Qian Wang

Zhanxiang Wang

Yi Wang

Jingyu Wang

Haibin Wang

Kwua-Yun Wang

Jinheng Wang

Jue Wang

Lin Wang

Lili Wang

Lei Wang

Mongheng Wang

Meilin Wang

Chun-Meng Wang

Jinn-Ling Wang

Yanzhou Wang

Zhentian Wang

Fuzhou Wang

Xiang Wang

Yong Wang

Ying Wang

Yuanjun Wang

Yi-Chian Wang

Xi Wang

Qiong Wang

Boliang Wang

Bei Wang

Han Wang

Tengfei Wang

Shang-Yu Wang

Andreas Wannhoff

Aaron Ward

Joanna Wardlaw

Peter Warnke

Dionysios Watson

Alexandra (Sasha) Webb

Rebecca Weddle

Jochen Wedemeyer

Piotr Wegrzyn

Liang Wei

Changyong Wei

Cun-Sheng Wei

Huiling Wei

Xian-Zhao Wei

Miriam Weinberger

Björn Weiß

Robert Weiss

Gregory Weitsman

Zhi Wen

Bixiu Wen

Ying Wen

Xuerong Wen

Lebin Wen

Junping Wen

Xisheng Weng

Hong Weng

Kai Wermker

Peter Whittaker

Matheus Wiest

Mohamed Wifi

Niels Wiinberg

Regan Williams

Craig Williams

James Willig

Henry Wilson

Nay Win

Kk Winer

Alexander Winter

Trisha Wise-Draper

Paula Witt-Enderby

Andrea Witzel

Wiktoria Wojciechowska

Alexandra Wolanskyj

Wong Won Fen

Jonathan Wong

Kelvin Wong

Mandy Wong

Catherine Wong

Raymond Wong

Jiong Wu

Jianyong Wu

Ziheng Wu

Fengchun Wu

Kerui Wu

Qi-Jun Wu

Shengjie Wu

Mei-Yi Wu

Jin-Ming Wu

Jian Wu

Kongming Wu

Jing Wu

Zunyou Wu

Shasha Wu

Wen Wu

Ruey-Meei (Robin) Wu

Te-Chang Wu

William Wu

Yingli Wu

Yen-Wen Wu

Jiang Wu

Wei Wu

Fangrui Wu

Deng-Chyang Wu

Jianguo Wu

Cho-Kai Wu

Yinyuan Wu

Jau-Ching Wu

Wojciech Wysocki

Xin Xia

Shuang Xia

Tingting Xia

Jie Xiang

Wen-Jun Xiao

Fei Xiao

Haiyan Xiao

Lanbo Xiao

Youping Xiao

Li Xiaoyan

Shiqi Xie

Shang Xie

Zhongqiu Xie

Wei Xie

Wuxiang Xie

Yuchao Xie

Liyi Xie

Haiting Xie

Jing Xie

Fei Xie

Lijun Xie

Wei Xiong

Yixiang Xu

Mafei Xu

Jinrui Xu

Jing Xu

Huajun Xu

Jiejie Xu

Min Xu

Biao Xu

Meng Xu

Peng Xu

Zhi-Xiang Xu

Haifei Xu

Haineng Xu

Beibei Xu

Shidong Xu

Lichen Xu

Jiru Xu

Jin-Fu Xu

Liang Xu

Gelin Xu

Ying Xu

Lihua Xu

Hidetake Yabuuchi

Santosh Yadav

Vishal Yadav

Lakshmi Narayana Yaddanapudi

Ajay Yadlapati

Javed Yakoob

Can Yaldiz

O Yaldizli

F. Firat Yalniz

Jason Yam

Ryotaro Yamada

Takamune Yamaguchi

Hiroko Yamashita

Shu-Ichi Yamashita

Qiang Yan

Jinchuan Yan

Bin Yan

Shenqiang Yan

Shirley Yan

Nachen Yang

Jingyun Yang

Yangfan Yang

Chun Yang

Hung-Chih Yang

Yanyan Yang

Yong-Qing Yang

Ju Yang

Mu Yang

Lingyao Yang

Haichun Yang

Jiao Yang

Jin Yang

Feng Yang

Rongqiang Yang

Yinmo Yang

Yongping Yang

Xiaoqin Yang

Xiaofang Yang

Seokjeong Yang

Ju Dong Yang

Cheng-De Yang

Dajun Yang

Xianrui Yang

Yoshihiko Yano

Lu Yao

Fan Yao

Wenlong Yao

Qing Yao

Naoki Yashimura

Hiroshi Yatsuhashi

Burcu Balam Yavuz

Ilhan Yaylim

Mehmet Fatih Yazar

Seyhan Yazar

Junna Ye

Tao Ye

Dingwei Ye

Cong Ye

Siva Harsha Yedlapati

Cumhur Yegen

Chih-Fan Yeh

Chuan-Min Yen

Chueh-Chuan Yen

Can Yerebakan

L Yessayan

Sha Yi

Bin Yi

Jing Yi

Eren Yıldız

Mehmet Tugrul Yilmaz

Jianyi Yin

Wen Yao Yin

Gui-Shuang Ying

Yanping Ying

Inomata Yino

Michelle Yip-Schneider

Hitoshi Yokoyama

Kunio Yokoyama

Jc Yombi

Tuck Yean Yong

Tuck Leong Yong

Li Yong

Yean Yong

Tatsuya Yoshida

Kengo Yoshimitsu

Taichiro Yoshimoto

Masafumi Yoshimura

Peng You Fan

Zhongxun Yu

Zhe Yu

Xiaofei Yu

Miao Yu

Minbin Yu

Hsien-Chung Yu

Meifang Yu

Kuang-Hui Yu

Pengfei Yu

Bin Yu

Shan Yu

Huang-Ping Yu

Ming-Lung Yu

Xuan Yu

Xiaojia Yu

Wen-Chung Yu

Xiaobin Yu

Tung-Min Yu

Yunyong Yu

Yao Yu

Bo Yuan

Peng Yuan

Wen Yuan

Jinhuan Yue

Liu Yueju

Siu Tsan Yuen

Zeng Yueqin

Murat Yuksel

Harun Yuksel

Ali Zaid

Gisele Zanca

Keivan Zandi

Philipp Zanger

Eva Zapata

Ben Zarzaur

John Zaunders

Przemyslaw Zdziarski

Maria Soledad Zegpi

Marko Zelić

Xavier Zendjidjian

Xiangpei Zeng

Hongwu Zeng

Ling-Li Zeng

Xiang Zeng

An Zeng

Qi Zeng

Gianpaolo Zerbini

Claudia Zerbini

Yiqiang Zhan

Yiyang Zhan

Minggang Zhang

Xiaoyong Zhang

Youjie Zhang

Wenwen Zhang

Xiao Dong Zhang

Jinqiang Zhang

Wan-Guang Zhang

Yongchun Zhang

Feng Zhang

Shijia Zhang

Deqiang Zhang

Hongxing Zhang

Lei Zhang

Gang Zhang

Chen Zhang

Zhuzhu Zhang

Yanqing Zhang

Jiming Zhang

Hui Zhang

Li Zhang

Guiming Zhang

Qinhong Zhang

Song Zhang

Yue Zhang

Zhongheng Zhang

Huan Zhang

Changqing Zhang

Xianwei Zhang

Wen-Na Zhang

Wei Zhang

Dongze Zhang

Hong Zhang

Liman Zhang

Jing Zhang

Heye Zhang

Lanjing Zhang

Melvyn Zhang

Yunlong Zhang

Yi Zhang

Xinhua Zhang

Guilian Zhang

Mei Zhang

Wenjie Zhang

Yan Zhang

C Zhang

Jiuquan Zhang

Min Zhang

Qi Zhang

Yuping Zhang

Yajia Zhang

Ma Zhanlong

Shilin Zhao

Chuntao Zhao

Xuna Zhao

Hong Zhao

Dongxin Zhao

Shangang Zhao

Jing Zhao

Xuan Zhao

Qinglan Zhao

Wei Zhao

Mary Zhao

Zhi-Jing Zhao

Hongxin Zhao

Fei Zhao

Yong Zhao

Zhigang Zhao

Jiuliang Zhao

Jia-Guo Zhao

Mingrui Zhao

Juan Zhao

Meng Zhao

Zibo Zhao

Yu-Kun Zhao

Jin Zhao

Jun Zhao

Sihai Zhao

Zeng Zhaochong

Guo-Qing Zheng

Minghua Zheng

Xiaoxu Zheng

Wang Zheng

Ce Zheng

Gen Zheng

Zhe Zheng

Jine Zheng

Linghua Zheng

Fang Zheng

Xingnan Zheng

Jian Zhong

Rui Zhong

Baoliang Zhong

Diansheng Zhong

Wen Zhou

Xianxiao Zhou

Bing Zhou

Guoying Zhou

Dong Zhou

Kehua Zhou

Huacheng Zhou

Yunyun Zhou

Yi Zhou

Dongsheng Zhou

Xu-Jie Zhou

Cindy Zhou

Jun Zhou

Fengqiong Zhou

Dang-Xia Zhou

Fen Zhou

Weiying Zhou

Yimin Zhu

Yongjin Zhu

Xiaodong Zhu

Chengcheng Zhu

Yonghong Zhu

Chuhong Zhu

Heming Zhu

Lucheng Zhu

Zhe Zhu

Zezhang Zhu

Yong-Tong Zhu

Jun Zhu

Xun Zhuang

Francesca Zimetti

Nicolle Zimmermann

Ljubo Znaor

Min Zong

Giacomo Zoppini

Juan Zou

Alauldeen Mudhafar Zubair

Haseeb Zubair

A Zuchetto

Stéphane Zuily

Zehua Zuo

Anna Zygogianni

Sa’Ed Zyoud

